# Thermodynamics of Potential CHO Metabolites in a Reducing Environment

**DOI:** 10.3390/life11101025

**Published:** 2021-09-29

**Authors:** Jeremy Kua, Alexandra L. Hernandez, Danielle N. Velasquez

**Affiliations:** Department of Chemistry & Biochemistry, University of San Diego, San Diego, CA 92110, USA; alexandrahernandez@sandiego.edu (A.L.H.); dvelasquez@sandiego.edu (D.N.V.)

**Keywords:** origin of life, proto-metabolism, chemical evolution, thermodynamics, prebiotic chemistry

## Abstract

How did metabolism arise and evolve? What chemical compounds might be suitable to support and sustain a proto-metabolism before the advent of more complex co-factors? We explore these questions by using first-principles quantum chemistry to calculate the free energies of CHO compounds in aqueous solution, allowing us to probe the thermodynamics of core extant cycles and their closely related chemical cousins. By framing our analysis in terms of the simplest feasible cycle and its permutations, we analyze potentially favorable thermodynamic cycles for CO_2_ fixation with H_2_ as a reductant. We find that paying attention to redox states illuminates which reactions are endergonic or exergonic. Our results highlight the role of acetate in proto-metabolic cycles, and its connection to other prebiotic molecules such as glyoxalate, glycolaldehyde, and glycolic acid.

## 1. Introduction

The extant metabolism of living systems is complex. However, at its core [1], represented by the tricarboxylic acid (TCA) cycle and its chemical cousins, the metabolites consist of only a small subset of molecules containing the elements carbon, hydrogen, and oxygen. Present metabolism is highly regulated. Specific biochemical reactions are catalyzed by specialized enzymes with help from a variety of other co-factor molecules. However, at the dawn of life’s origin, before the complex machinery of biochemical machinery evolved to support and sustain metabolism, what might a *proto*-metabolism look like?

As pointed out by Pross [2] in this Special Issue, “all material systems are driven towards more persistent forms.” In the context of which specific molecules will persist (in equilibrium or at steady state) and thereby contribute to the small subset utilized by proto-metabolism, both thermodynamics and kinetics will play a role. Our present study will only focus quantitatively on the thermodynamics, while making qualitative reference to kinetics. This is partly due to limitations in our present methodology (see Methods section), partly to keep the problem at hand tractable, but also partly because we think a survey of the thermodynamics is an interesting tale in its own right.

Autocatalysis lies at the heart of persistence. If a molecule can catalyze its own production faster than its competitors, it will persist (maintaining a non-zero and possibly significant concentration as a function of time)—until its “food” runs out or a parasitic reaction shunts it out of the main network. For a broad overview of autocatalytic networks and their role at life’s origin, we recommend the review by Hordijk and Steel [3] in this journal. Our present study focuses on just one class of reaction cycles: those that produce a net two-carbon molecule from one-carbon substrates. Extant life on our planet ultimately depends on fixing CO_2_ as its carbon source to build biomass. While proto-life on the early Earth may have had a variety of carbon sources, the ability to incorporate C_1_ molecules into larger structures remains fundamental for a self-sustaining system to emerge [4]. Thus, incorporating C_1_ + C_1_ → C_2_ into a sustaining cycle is fundamental.

In the absence of complex biomolecular enzymes, high-energy activation barriers are unlikely to be traversed at temperatures conducive to life. We can narrow down potential cycles by looking at thermodynamics: feasible chemistry will exclude overly endergonic reactions, while an overly exergonic reaction mid-cycle would require a subsequent endergonic step, thereby narrowing the possibilities further. We first examine the core TCA cycles and its chemical cousins building on work by Braakman and Smith [1], focusing on the reverse (i.e., reductive) direction in the absence of any enzymes or co-factors. (For recent reviews of non-enzymatic metabolic reactions including the potential role of a reverse TCA cycle at the origin of life, see Muchowska et al. [5] and Tran et al. [6]; for a review on the energetics of biomolecular synthesis, see Amend et al. [7])

We then expand the scope of potential metabolites beyond those used by extant life to a wider range of CHO compounds. We are not the first to examine this wider scope. Morowitz et al. suggested a set of 153 compounds ranging from C_1_ to C_6_ (70 in the C_1_ to C_4_ range) based on simple rules of thumb [8]. Meringer and Cleaves extended and refined this set using structure-generation methods from existing molecular databases to examine if the metabolites of the reverse TCA cycle are “optimal” [9]. (Their conclusion: maybe not.) Zubarev et al. examined the thermodynamics of a potential reverse TCA “supernetwork” (175 molecules, 444 reactions); they use computationally faster (but less accurate) semi-empirical methods to calculate the Gibbs free energies; and they conclude that there exist families of TCA-like reactions with similar energetic profiles [10].

Our present research complements these earlier “bird’s eye view” studies by focusing on the details of the smaller C_1_ to C_4_ species. We calculate the (aqueous) Gibbs free energy of each species using first-principles quantum chemistry. Our method is more computationally-intensive (but not terribly so) and matches well with experimental data where available. To map the thermodynamic landscape, our set of 211 C_1_ to C_4_ compounds is much wider than those generated by the “Morowitz rules” [8] because we include highly reduced compounds that are unlikely to be metabolites. Our present study also limits the oxidizing and reducing agents to CO_2_ and H_2_, respectively. We do this to establish baseline data for future work.

While our data allow for a wide range of analyses, this paper will pick out a few key examples to illustrate how one might build a proto-metabolic cycle, why paying attention to redox states is important, and why particular molecules may be keystone species, thermodynamically-speaking. In particular, we will (1) explain why the C_1_ + C_1_ → C_2_ reaction must be incorporated into a cycle, (2) examine the close relationship between carbonyl compounds as potential metabolites, (3) suggest reasons for the centrality of acetate in core metabolic cycles, and (4) discuss specific reactions and molecules that could participate in proto-metabolic cycles in the absence of highly specific catalysts. In no way do we discount the importance of other compounds beyond the limited set we have calculated (compounds containing nitrogen, sulfur and phosphate; metal ions/clusters) that can act as potential catalysts and cofactors; nor will we discuss the important question of flows in non-equilibrium thermodynamic systems. Ongoing research in our laboratory explores these extensions but they are beyond the scope of the present work.

## 2. Materials and Methods

To construct our thermodynamic maps, we have established a protocol to calculate the *relative* aqueous free energy of each molecule using quantum chemistry. This protocol has been described in detail in our previous papers (most recently in [11]) and shows good agreement with available experimental results for CHO systems [12,13,14]. Herein, we briefly summarize the protocol and point out some of its limitations, reproducing some portions of text in our previous work [11] for clarity and reading ease.

The structure of each molecule is optimized and its electronic energy calculated at the B3LYP [15,16,17,18] flavor of density functional theory with the 6-311G** basis set. To maximize the probability of finding global minima, multiple conformers are generated using molecular mechanics (OPLS force field with water as a solvent) [19]. The optimized structures are embedded in a Poisson–Boltzmann continuum to calculate the aqueous solvation contribution to the free energy. While this does not provide a specific concentration, it assumes a dilute solution such that the electrostatic field generated by a neighboring solute molecule is effectively screened by the water solvent. One can consider all solutes to have the same relative concentrations in our calculations.

Zero-point energy corrections are included based on the calculated analytical Hessian. We apply the standard temperature-dependent enthalpy correction term (for 298.15 K) from statistical mechanics by assuming translational and rotational corrections are a constant times *kT*, and that low-frequency vibrational modes generally cancel out when calculating enthalpy differences. So far, this is standard fare.

Entropic corrections in aqueous solution are more problematic [20,21,22]. Changes in free energy terms for translation and rotation are poorly defined in solution due to restricted complex motion, particularly as the size of the molecule increases (thus increasing its conformational entropy). Free energy corrections come from two different sources: thermal corrections and implicit solvent. Neither of these parameters is easily separable, nor do they constitute all the required parts of the free energy. We follow the approach of Deubel and Lau [23], assigning the solvation entropy of each species as *half* its gas-phase entropy (calculated using standard statistical mechanics approximations similar to the enthalpy calculations described above), based on proposals by Wertz [24] and Abraham [25] that upon dissolving in water, molecules lose a constant fraction (~0.5) of their entropy.

When put to the test by first calculating the equilibrium concentrations in a self-oligomerizing solution of 1 M glycolaldehyde at 298 K, our protocol fared very well compared to subsequent NMR measurements [14]. That being said, our protocol does show systematic errors when calculating barriers, but since quantitative kinetics is not part of the present study, this is not an issue. When extended to include nitrogen-containing species, our protocol still does well but systematic errors do crop up for specific functional groups [26]. Going to a higher level of theory and/or including dispersion corrections does not improve the results (see Table S1); this may seem surprising but quantum chemistry is about error cancellation, and our protocol (with its foibles) seems to work well, at least for the compounds in this study.

The *relative* aqueous free energies, designated *G_r_*, are calculated with respect to three reference molecules: H_2_, H_2_O, and H_2_CO_3_. We use H_2_CO_3_ instead of CO_2_ because it allows a direct comparison with experimentally-derived thermodynamic data from Alberty [27]. Thus, if *G* is the free energy calculated from quantum chemistry, then the formation reaction of glycolaldehyde is 2 H_2_CO_3_ + 4 H_2_ → C_2_H_4_O_2_ + H_2_O, and *G_r_*(glycolaldehyde) = *G*(C_2_H_4_O_2_) + *G*(H_2_O) − 2 *G*(H_2_CO_3_) − 4 *G*(H_2_). In this study, free energy corrections for concentration differentials among species (to obtain chemical potentials) are not included.

We have calculated all compounds in their neutral form. Thus, acetic acid is calculated rather than acetate, because this gives much better results in comparison to experimental data as discussed in the Results section, where we compare our calculations of both the neutral and anionic forms for the TCA cycle. (Additionally, our protocol was established and validated only for neutral compounds.) Additional comparisons of neutral versus anions for other cycles are provided in Tables S2–S4. The protocol used for calculating anions is similar to the neutral, except for the addition of diffuse functions to the basis set of anions, a standard procedure in quantum chemical calculations.

Our set of calculated compounds includes all CHO molecules from C_1_ to C_4_ with carbon-carbon backbones (except alkynes). Select compounds with oxygen in the backbone (ethers, esters, anhydrides) are included, although non-exhaustively (they are less stable than their carbon-backbone isomers). For each unique molecular species, we report only the free energy of the most stable conformer. In a small number of cases, this may be the enol or the hydrate. For compounds with one chiral center, the biologically relevant one was calculated; for two chiral centers, the most stable diastereomer is reported. The list of compounds explicitly mentioned in the Results section and their corresponding *G_r_* values can be found in Appendix A. The full list is provided in Table S5). A graphical comparison of our calculated *G_r_* values to experimentally-derived ones is also shown in Appendix A.

Assigning H_2_, H_2_O, and H_2_CO_3_ as reference states allows us to quickly and easily visualize a map of the energy landscape for the myriad reactions that can take place. For a chemical reaction, the difference in free energies will be designated Δ*G*, calculated as *G_r_*(products)–*G_r_*(reactants). For example, Δ*G* for the conversion of acetic acid into pyruvic acid is simply *G_r_*(pyruvic acid)–*G_r_*(acetic acid), since the reference state molecules (H_2_, H_2_O, H_2_CO_3_) by definition have *G_r_* values of zero.

## 3. Results

Our results and discussion are organized as follows: (1) First, we provide further validation of our methods by comparing our calculated Δ*G* values against experimental data for the TCA cycle. (2) Next, we examine the free energy profiles of four core CO_2_-fixation cycles in Braakman and Smith [1]. (3) Then, we discuss why embedding C_1_ + C_1_ → C_2_ in a cycle is fundamental using the example of formaldehyde oligomerization. (4) This leads naturally to our exploring a wider scope of reactions and cycles that might have played a role in proto-metabolic systems. (5) Finally, in the Discussion section, we take a bird’s eye view of the overall thermodynamic picture with an eye on redox states.

### 3.1. TCA Cycle: Comparison to Experimentally-Derived Data

Using experimentally-measured equilibrium constants, Alberty derived standard free energies of formation for a range of biochemical compounds [27]. Not surprisingly, there is a relatively good match when comparing the Gibbs free energies of reaction from Alberty’s Table 4.10 (pH 7, 298.15 K, 0.25 M ionic strength, 1 M solute) to data provided from a standard biochemistry textbook [28], for the TCA cycle as shown in Table 1. The main discrepancy is Step 1, where Alberty has a more exergonic reaction by 3.2 kcal/mol. As a result, Alberty’s net cycle is overall 3 kcal/mol more exergonic than the textbook value. Note that Alberty *assigns* Δ*G* = 0.0 in Step 6 to match the experimental data when attempts to derive free energies for the enzyme-bound FAD proved unsatisfactory.

How do our quantum calculations compare? We do not explicitly calculate the co-factors CoA, NAD^+^/NADH and ADP/ATP. Therefore, to correct for their inclusion, we recalculated Alberty’s Δ*G* values for each reaction in the absence of these co-factors to find empirical corrections, which are: −7 kcal/mol for AcCoA → CoA + acetate; −9 kcal/mol for NAD^+^ → NADH; and +7 kcal/mol for ADP + P_i_ → ATP. Both Alberty and the textbook split Step 5 into two separate reactions, but since we do not explicitly calculate succinyl-CoA as separate from succinate, we combined these into a single step.

Our results show some differences compared to the experimental data. We calculate larger energy changes for the hydration/dehydration sequence in Steps 2 and 3. For the NAD^+^/NADH couple, our Step 8 is slightly more endergonic, while our Steps 4 and 5 are more exergonic. Opposite to Alberty, our Step 1 Δ*G* value and net cycle are both less exergonic than the textbook data. However, we are more interested in the free energy profiles in the absence of these co-factors, and to that data we now turn.

Table 2 shows Δ*G* calculated from five sources of data:

- Alberty’s Table 4.1 [27]: Δ_f_*G_i_′^0^* values (298.15 K, pH 7, zero ionic strength, 1 M solute) as these correspond to our quantum calculations that do not include ionic strength.
- The larger experimentally-derived dataset from eQuilibrator [29,30] that built on and expanded the Alberty data: Δ_f_*G_i_′^0^* values also at pH 7 and zero ionic strength.
- Our quantum calculated data for neutral molecules.
- Our quantum calculated data for anions (with the addition of diffuse functions in the basis set for all calculations, and adding bicarbonate as a reference compound).
- The group-additivity approach of Jankowski et al. [31]: Δ_f_*G′^0^* values (derived at zero ionic strength)

The reaction cycle has nine steps because we explicitly include oxalosuccinate as a distinct species. For the metabolite names, we use the abbreviations of Braakman and Smith in their depiction of the four core-cycles (to be discussed in the next section); name–abbreviation connections are found in Appendix A. H_2_ is the reductant in this system.

Not surprisingly, the Alberty and eQuilibrator data are very similar with the exception of OXS, where their Δ_f_*G_i_′^0^* values differ significantly. This leads to the 7–8 kcal/mol uphill and downhill differences in Steps 4 and 5. However, the overall net cycles are similar at +44 kcal/mol. (With no co-factors the *oxidative* TCA cycle is rather endergonic!) The most endergonic step is the oxidation of SUC to FUM, and in the absence of the enzyme-bound-FAD complex, the raw cost of breaking two C–H bonds to form a π bond between the carbons and one H–H bond is expected to be approximately +25 kcal/mol.

Our quantum calculations for the neutral molecules are not too different (they match Alberty slightly better than eQuilbrator) and the overall cycle is +45 kcal/mol. This gives us confidence that our calculated Δ*G* values match reasonably well with experimental-derived data. In contrast, if we calculate all these compounds as anions (carboxylates) instead of the neutral COOH groups, there are significant differences in more than half the steps, although the overall cycle is not too different at +50 kcal/mol. (Using a higher level of theory does not improve the result; see Table S1.)

Experimentally-derived data are limited to compounds of biochemical interest. However, our goal is to expand the set of compounds to explore closely related compounds not utilized by extant biochemistry. Hence, we also used the group additivity method of Jankowski et al. [31] to calculate the reactions in this cycle. (Note that eQuilibrator also utilizes some group additivity in its calculations [30].) In this case, the Jankowski scheme does poorly when compared to Alberty. The overall cycle is +15 kcal/mol primarily due to underestimating the endergonicity of the oxidation steps (4, 7 and 9).

For the rest of the Results section, we will not be discussing the quantum calculations using anions since their Δ*G* values do not match well with experiment. (These, along with additional Jankowski and eQuilibrator data are included in Supplementary Materials for the interested reader.) Additionally, because the Alberty dataset is limited, we will compare our quantum calculations to eQuilibrator data where available.

### 3.2. Thermodynamics of Core Cycles for CO_2_ Fixation

**Figure 1:**
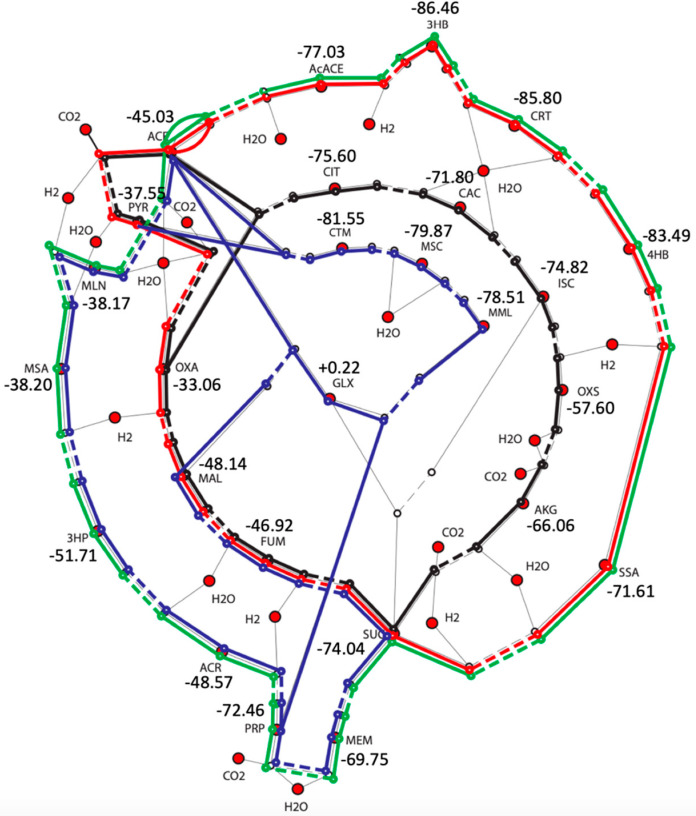
Metabolites and *G_r_* values (in kcal/mol) for the four core metabolic cycles in Braakman and Smith.

Since the oxidative TCA cycle is net endergonic, this provides an opportunity for exergonic CO_2_ fixation by running the cycle in reverse. Figure 1 shows the four core cycles: TCA (in black), dicarboxylate/4-hydroxybutyrate (DC/4HB in red), 3-hydroxypropionate bicycle (3HP, in blue), and 3HP/4HB (in green). Figure 1 is adapted (under the Creative Commons license agreement) from Figure 6 in Braakman and Smith [1], where we have added the *G_r_* values (in kcal/mol) from our quantum calculations for each metabolite. 

As described in the Methods section, all *G_r_* values are with respect to the reference molecules H_2_, H_2_O and H_2_CO_3_. This allows us to quickly calculate Δ*G* for any reaction of interest in these cycles. For example, oxaloacetatic acid (OXA) in the *reductive* TCA cycle is first reduced to malic acid (MAL). (We use the “acid” names rather than the anion names because our calculations are for the neutral form.) Δ*G* for this reaction is −48.14 + 33.06 = −15.08 kcal/mol. This reduction reaction is the exact opposite of its oxidative counterpart (Step 9 in Table 2). The overall cycle is exergonic with a net change of −45.03 kcal/mol (the exact opposite of +45.03 kcal/mol in Table 2). This value is also equal to the net chemical reaction of the cycle which converts two equivalents of CO_2_ into acetic acid.

2 H_2_CO_3_ + 4 H_2_ → ACE + 4 H_2_O (1)

Equation (1) is also the net reaction for the DC/4HB and 3HP/4HB cycles, and is a key example of incorporating the C_1_ + C_1_ → C_2_ reaction into a cycle.

In Figure 1, the *G_r_* value for ACE is −45.03 kcal/mol since Equation (1) represents the formation of ACE with all other molecules being reference states. For eQuilibrator data, the equivalent *G_r_* value for acetate is −44.63 kcal/mol (see Appendix A), which is exactly what you would expect given the net cycle in the oxidative direction from Table 2 is +44.63 kcal/mol.

#### 3.2.1. The Reductive TCA Cycle

**Figure 2:**
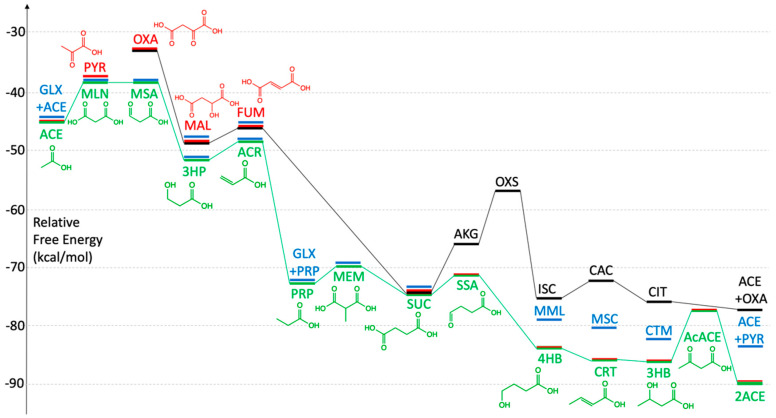
Reaction free energy profiles for the four core cycles.

Let us examine the free energy changes for each step along the reductive TCA cycle by following the black lines in Figure 2. The reaction sequence is as follows:

1. OXA → MAL (reduction)—exergonic,
2. MAL → FUM (dehydration)—mildly endergonic,
3. FUM → SUC (reduction)—very exergonic,
4. SUC → AKG (formal addition of CO_2_ + H_2_)—endergonic,
5. AKG → OXS (formal addition of CO_2_)—endergonic,
6. OXS → ISC (reduction)—exergonic,
7. ISC → CAC (dehydration)—mildly endergonic,
8. CAC → CIT (re-hydration)—mildly exergonic, and
9. CIT → OXA + ACE (splitting reaction)—mildly exergonic.

The two mildly endergonic steps (2 and 7) are dehydration reactions. The two main endergonic steps (4 and 5) both involve oxidation because CO_2_ (with a +4 oxidation state of carbon) is added. The first of these also adds an equivalent of the reductant H_2_, i.e., a net change in oxidation of 2 units from SUC (+2) to AKG (+4). The second does not include H_2_ and changes the oxidation state by 4 units: AKG (+4) to OXS (+8). Each step is ~8 kcal/mol uphill, and both steps occur in succession. Note that the first addition involves carboxylation at the terminus, while the second adds a branch to the backbone.

In contrast, the reduction steps (1, 3 and 6) are significantly downhill (Δ*G* of −15, −17 and −27 kcal/mol for OXA → MAL, OXS → ISC and FUM → SUC, respectively), and are the reason why the net cycle is overall exergonic. The final step splitting CIT (+6) to produce ACE (0) and regenerate OXA (+6) is mildly exergonic; OXA has the same overall oxidation state as CIT.

Could the reductive TCA cycle play a key role in prebiotic proto-metabolic systems? It is unclear. Having two successive endergonic reactions would require an oxidizing co-factor strong enough to compensate for the unfavorable uphill ~16 kcal/mol. The question of whether OXA could play the key role as the “recycled” molecule also poses problems given its kinetic instability to β-decarboxylation (and forming PYR). It is also unclear that the specific larger C_5_ and C_6_ compounds of the reductive TCA would be present in sufficient quantities to participate in a sustaining primordial cycle. 

#### 3.2.2. The 3HP/4HB Cycle

The 3HP/4HB cycle (green lines in Figure 1 and Figure 2) mirrors the TCA cycle but only involves the smaller C_2_ to C_4_ metabolites. The net reaction is Equation (1) and the free energy change of the overall cycle is −45.03 kcal/mol. The “recycled” molecule is ACE itself. Let us consider the reaction steps in detail.

1. ACE → MLN (formal addition of CO_2_)—endergonic,
2. MLN → MSA (acid-to-aldehyde reduction)—little change,
3. MSA → 3HP (reduction)—exergonic,
4. 3HP → ACR (dehydration)—mildly endergonic,
5. ACR → PRP (reduction)—very exergonic,
6. PRP → MEM (formal addition of CO_2_)—endergonic,
7. MEM → SUC (methyl shift)—mildly exergonic,
8. SUC → SSA (acid-to-aldehyde reduction)—little change,
9. SSA → 4HB (reduction)—exergonic,
10. 4HB → CRT (dehydration)—mildly exergonic, which seems odd,
11. CRT → 3HB (re-hydration)—mildly exergonic,
12. 3HB → AcACE (oxidation)—endergonic, and
13. AcACE → 2 ACE (splitting reaction)—exergonic.

The first CO_2_ addition takes place in the very first step at the α-carbon, converting ACE to MLN. This makes sense; highly oxidized CO_2_ has a very positive carbon so we expect it to add to the more negative (or least positive) carbon on ACE, in this case the methyl group. For this oxidation step, Δ*G* = +7 kcal/mol. Note, however, that in the absence of catalysts, the addition of CO_2_ will have a high barrier.

The second step is a reduction of carboxylic acid to aldehyde to form MSA, and unlike reductions we have seen in the TCA cycle, it is energetically neutral. We will expand on this feature later, but for now, note that it involves a condensation (releasing H_2_O) in concert with the addition of H_2_. In contrast, the reactions we examined in the TCA cycle only involved H_2_ addition (ketone to alcohol, or the very exergonic alkene to alkane).

The next several steps MSA → 3HP → ACR → PRP are similar to the OXA → MAL → FUM → SUC sequence. However, when PRP is oxidized by an equivalent of CO_2_, branched MEM is produced rather than linear SUC. Once again, it makes sense to add CO_2_ to the least positive carbon, in this case the α-carbon of PRP (in terms of alternant “plus” and “minus” carbons in the chain of a polar compound). Interestingly, this reaction is only mildly endergonic (Δ*G* = +3 kcal/mol), noticeably less than previous CO_2_ incorporations we have considered thus far. We will see this again in several cases when CO_2_ is added to a carbon that is not bonded to oxygen (e.g., a methyl or methylene group).

Although exergonic, the isomerization of MEM to SUC whereby the methyl branch insinuates itself into the chain is unlikely to occur in a prebiotic milieu (not a problem for the evolved enzyme methylmalonyl-CoA mutase); and likely has a high barrier (uncatalyzed). Moving along, SUC → SSA → 4HB mirrors MLN → MSA → 3HP, although SUC → SSA is mildly exergonic instead of being energy neutral. You might expect dehydration of 4HB to be mildly endergonic, and it would be indeed if but-3-enoic acid was formed (see Section 3.4), but the formation of CRT also includes a double bond shift to the more stable isomer. Rehydration to 3HB then becomes mildly endergonic.

However, because 4HB/CRT/3HB have oxidation state −2, the cycle now requires an alcohol to ketone oxidation, uphill ~8 kcal/mol. Once zero-oxidation state AcACE is formed, it can split apart to two ACE molecules in a reaction resembling a reverse Claisen condensation; this reaction that recycles ACE is downhill (Δ*G* = −13 kcal/mol).

How does the 3HB/4HB cycle compare to TCA? It splits up the endergonic steps involving oxidative CO_2_ addition, the second being only mildly endergonic, which might be advantageous. However, the cycle over-reduces, thus requiring a third endergonic oxidation step before splitting and recycling ACE. Furthermore, converting branched MEM to SUC, although thermodynamically favorable, is likely to be kinetically inaccessible without highly specific enzymes or co-factors.

Compared to eQuilibrator, our *G_r_* values (in Figure 1) for the C_3_ species are quite similar with the exception of PRP: eQuilibrator *G_r_* values (in kcal/mol) are MLN (−39.42), MSA (−38.22), 3HP (−52.18), ACR (−49.53), PRP (−77.04). For PRP, our calculated *G_r_* is −72.46, a difference of over 4 kcal/mol. For the C_4_ species, our *G_r_* values are consistently more negative by ~2 kcal/mol with the exception of 3HB and 4HB: eQuilibrator *G_r_* values (in kcal/mol) are MEM (−67.76), SUC (−71.35), SSA (−69.51), 4HB (−77.78), CRT (−82.49), 3HB (−87.39), AcACE (−75.35). For 4HB, our calculated *G_r_* is −83.49, a difference of over 5 kcal/mol. For 3HB, our calculated *G_r_* is −86.46 which is less negative than eQuilibrator.

#### 3.2.3. The DC/4HB Cycle

The DC/4HB cycle (red lines in Figure 1 and Figure 2) utilizes the first half of the TCA cycle (OXA to SUC) and the second half of the 3HP/4HB cycle (SUC to AcACE). It also only involves the smaller C_2_ to C_4_ metabolites, and once again the net reaction is the same as Equation (1) and the overall cycle is −45.03 kcal/mol. However, it begins with ACE (and not OXA), and thus the recycled molecule is ACE itself.

The first two steps frontload the uphill oxidative incorporation of CO_2_ equivalents. ACE → PYR is analogous to SUC → AKG, in that “CO_2_ + H_2_” are added to the terminal carboxylic acid, and the oxidation state changes by +2. The second step adds CO_2_ to the least positive carbon, in this case the methyl of PYR (+2), to form OXA (+6) with an oxidation state change of +4 mirroring the AKG → OXS reaction. However, unlike SUC → AKG → OXS which is 16 kcal/mol uphill, ACE → PYR → OXA is only 12 kcal/mol uphill because the second step is less endergonic (Δ*G* = +5 kcal/mol). While this seems more feasible, the question is whether the first carboxylation of ACE to PYR is more or less favorable than to MLN. Thermodynamically, there is little difference (MLN is a mere 0.6 kcal/mol more stable), but kinetically things could be quite different depending on what primitive catalysts or co-factors might be present and whether H_2_ can easily be incorporated as a reductant in this first step. Answering this question is beyond the scope of this article (we will tackle the kinetics in future work). As previously mentioned, CO_2_ addition will have high barriers in the absence of catalysts, although our present work focuses quantitatively only on the thermodynamics.

The rest of the cycle was discussed earlier since it overlaps with the TCA and 3HP/4HB cycles. Once OXA is formed, it is mostly downhill all the way to 3HB except that over-reduction requires an uphill oxidation to AcACE prior to splitting apart into two ACE. Our calculated *G_r_* for PYR (−37.55) is marginally less negative than eQuilibrator’s value of −38.87 kcal/mol.

#### 3.2.4. The 3HP Bicycle

Unlike the previous three cycles, the 3HP bicycle (blue lines in Figure 1 and Figure 2) does not have the same net reaction. The first half of the cycle from ACE to PRP overlaps with the 3HP/4HB cycle, but then the path splits into two: (1) PRP can add GLX to access C_5_ compounds (MML, MSC, CTM) before splitting into ACE and PYR. (2) PRP can add CO_2_ to access the C_4_ compounds (MEM, SUC, FUM, MAL) before splitting into ACE and recovering GLX. Thus, the net reaction as shown in Equation (2) is

PRP + 2 H_2_CO_3_ + 2 H_2_ → ACE + PYR + 3 H_2_O (2)

and the net cycle is exergonic with a free energy change of −10.12 kcal/mol. While forming ACE from CO_2_ contributes −45.03 kcal/mol as expected, oxidizing PRP to PYR is −37.55 + 72.46 = +34.91 kcal/mol. Hence, the net −10.12 kcal/mol.

GLX (glyoxylic acid / glyoxalate) is the key intermediate in the bicycle. It is a highly oxidized (+4) C_2_ compound and a high-energy intermediate (*G_r_* = +0.22). This means that when employed as a reactant, it can drive a downhill reaction to form thermodynamically more stable products. However, recycling GLX comes at a cost. The killer step, in this case, is the oxidation of SUC to FUM (Δ*G* = +27.12 kcal/mol), part of the sequence PRP → MEM → SUC → FUM → MAL → ACE + GLX which is oxidative and endothermic +27.65 kcal/mol. This is balanced by the downhill sequence starting from ACE and involving the C_5_ compounds (Δ*G* = −37.77 kcal/mol), recycling ACE while converting GLX (+4) to PYR (+2), a net reductive reaction.

In the left half of Figure 2, there is a small gap separating the blue bars from the other bars, signifying the presence of GLX contributing *G_r_* = + 0.22 kcal/mol to the relative free energy. We will revisit the potential wider role of GLX in Section 3.4.

### 3.3. Back to Basics: Incorporating C_1_ + C_1_ → C_2_ into a Cycle

While Stanley Miller’s landmark experiment [32] demonstrated that the synthesis of amino acids was possible in a *highly* reducing atmosphere [33], the present scientific majority view as summarized by Kasting [34] is that CO_2_ rather than CH_4_ was the dominant C_1_ building block available. Whatever the prebiotic milieu may have been, even if a range of carbon-containing compounds was available, the growth and sustenance of a proto-metabolic cycle requires “food” molecules—likely to be C_1_, as this would deplete specific larger molecules in the cycle (via hydrolysis or other parasitic side-reactions) unless they are replenished by a C_1_ source.

The problem, however, is that the direct C_1_ + C_1_ → C_2_, even if thermodynamically feasible, is kinetically challenging. In CO_2_ (or HCO_2_H, CH_2_O, CH_3_OH), carbon carries a partial positive charge because of the more electronegative oxygen. Making a new C–C bond between two such entities requires traversing high activation barriers. In the absence of co-factors or catalysts that could significantly lower the barrier (or using prohibitively high reaction temperatures), regular and constant production of C_2_ (or larger) molecules would be very slow. While the carbon of CH_4_ carries a small partial negative charge, CH_4_ is generally unreactive to forming new C–C bonds (in the absence of metal-containing catalysts).

CO is an interesting case; its net dipole is close to zero although its carbon carries a small negative partial charge; but CO is much less stable (thermodynamically) than CO_2_ and unlikely to exist in large quantities over a long period of time. It could, however, play an important role in situ, generated transiently from CO_2_ in the presence of H_2_. Using our reference states, this reaction as shown in Equation (3) is:

H_2_CO_3_ + H_2_ → CO + 2 H_2_O (3)

From our quantum calculations, *G_r_*(CO) = +3.33 kcal/mol, and thus this reaction is endergonic. (In contrast, formation of the other C_1_ compounds from CO_2_ and H_2_ is exergonic, see the Discussion section.) This reaction could play a role in CO_2_-fixation steps, as we have seen in the previous section, where several reactions involved the formal addition of “CO_2_ + H_2_” such as ACE (0) → PYR (+2).

The cycles we examined in the previous section all involve adding CO_2_ to a species that is C_2_ or larger. Why is this kinetically more feasible? A polar organic molecule, where one of the carbons carries a (significant) partial positive charge usually also contains neighboring carbons that are less positive or even have a (slight) partial negative charge. Addition of CO_2_ can be “directed” towards the less positive carbon of this larger molecule with a lower barrier—an *umpolung*-like “strategy” if you will. This provides a feasible way to incorporate CO_2_ without the cost of direct C_1_ + C_1_ addition.

**Figure 3:**
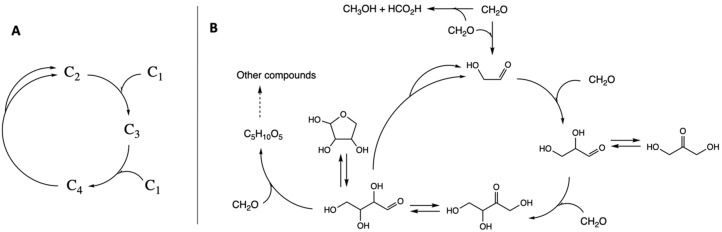
(**A**) Simplest cycle. (**B**) Key features of the CH_2_O oligomerization cycle.

However, how is the cycle created? At some point, a larger molecule would have to split into smaller entities (but not C_1_ as this would simply be a reverse addition). Hence, the simplest cycle that can be generated involves a C_2_, C_3_, and a C_4_ species, with the C_4_ able to split into two C_2_ species, as shown in Figure 3A.

We illustrate this with a formaldehyde oligomerization cycle, part of the formose reaction [35], as shown in Figure 3B. In previous work [13], we calculated the free energy profiles for CH_2_O oligomerization and explored both the thermodynamic and kinetic features of this cycle, which we now summarize briefly. Experimentally the reaction starting with solely CH_2_O has an induction period because the direct dimerization of CH_2_O to form glycolaldehyde (GA) has a very high barrier (recall our earlier discussion of why direct C_1_ + C_1_ → C_2_ is kinetically challenging). Once the C_2_ species is formed, however, aldol addition of CH_2_O proceeds rapidly (under appropriate experimental conditions) to generate a wide range of CHO-containing molecules, bypassing the direct C_1_ + C_1_.

The cycle has several interesting features. Aldol additions of CH_2_O (hydrate) are exergonic (−6 kcal/mol, on average). The C_2_ + C_1_ → C_3_ kinetically favors glyceraldehyde (GLA), which may subsequently isomerize (via an enol intermediate) to thermodynamically more stable dihydroxyacetone (DHA). The kinetically favored C_3_ + C_1_ → C_4_ converts GLA into the ketone (erythrulose), which is thermodynamically more stable than its aldehyde isomer (erythrose). Erythrose can potentially cyclize, add further CH_2_O units, or undergo a reverse aldol (C_4_ → C_2_ + C_2_) to regenerate GA. The splitting reaction is marginally endergonic (+2 kcal/mol). The overall cycle is exergonic by −7 kcal/mol.

We note three other interesting things about this system: (1) Active species in the cycle are in equilibrium with “off-cycle” compounds, which together form a “pool” of interconnected compounds—a feature that could stabilize the cycle and provide a point of regulatory control [36]. (2) Because the C_2_ “recycling” species (GA) and the C_1_ “food” species (CH_2_O) are both at oxidation state zero, additional redox reactions are not required, thereby simplifying the situation. (3) Cannizzaro reactions (such as 2 CH_2_O + H_2_O → HCO_2_H + CH_3_OH) can be parasitic on the cycle, but also provide access to compounds with a wider range of oxidation states; and experimentally the formose reaction generates a range of carboxylic acids [37], including those found in extant biochemistry. 

This sets the stage for us to explore alternative cycles that are thermodynamically favorable, but possibly more feasible kinetically than the extant cycles we have analyzed in the previous section.

### 3.4. Alternative Cycles: A Brief Exploration

In extant biochemical cycles, ACE (overall zero oxidation state) is the key recycled C_2_ species. The main goal of this section is to explore if this role can instead be played by one of its chemical cousins: ethanal (−4), glycolaldehyde (−2), glycolic acid (+2), or GLX (+4). We will also allude to CH_2_O as a potential crossover C_1_ food species, and we will see that intersecting cycles allow for a variety of possibilities. Before we embark, some words of caution. We remind the reader that our quantum calculations only provide thermodynamic data of the CHO metabolites; they do not take into account kinetics or even the presence of co-factors (such as sulfur-containing compounds) to tune the thermodynamics. We also chose to highlight a very limited set of specific examples that we think are interesting out of many possibilities. One should not over-interpret our results and fall into the trap of believing Kipling-esque *Just So* stories.

#### 3.4.1. Minor Modifications to the 3HP/4HB Cycle

**Figure 4:**
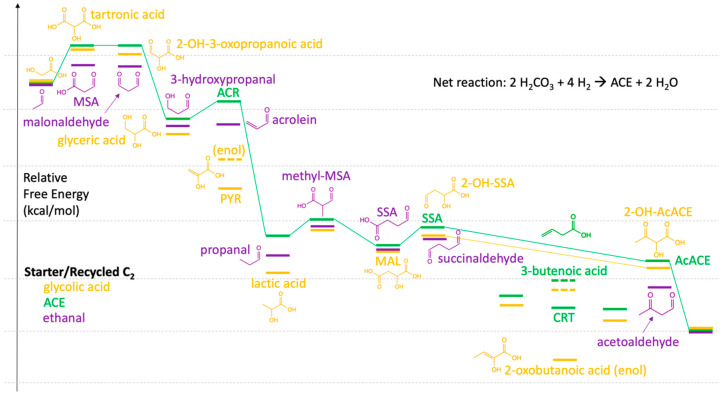
Relative energy profiles of modified 3HB/4HP cycles utilizing glycolic acid and ethanal.

Before jumping into alternative C_2_ species, one drawback to the extant 3HP/4HB cycle as discussed in Section 3.2.2 is over-reducing the carbon moiety, thereby requiring an endergonic step to generate AcACE before it splits into two ACE. A straightforward way to avoid this: If SSA is not reduced to 4HB but instead undergoes a thermodynamically favorable aldehyde-to-ketone isomerization (via the enol), AcACE can be formed directly from SSA, as shown in the right half of Figure 4 (long green line) We would expect this direct SSA → AcACE conversion in the absence of any reducing agents.

However, the preceding reduction step, SUC → SSA, requires a reducing agent, and it may not be feasible to halt reduction of the carboxylic acid at the aldehyde depending on the reducing agent and the reaction conditions. If 4HB is formed, we would expect dehydration to result first in but-3-enoic acid (see Figure 4) followed by rehydration to 3HB, but now the endergonic oxidation must be carried out to form AcACE. Considering the pool of compounds in equilibrium, certainly CRT is in the mix as an isomer of but-3-enoic acid (*G_r_* = −80.19); an isomer of 3HB and 4HB is 2-hydroxybutanoic acid (*G_r_* = −82.79); and 2-oxobutanoic acid (*G_r_* = −68.75) is an isomer of SSA and AcACE. The extant 3HP/4HB cycle utilizes the thermodynamically favorable isomers, but this may be less kinetically feasible for a proto-metabolic cycle that avoids or minimizes uphill steps.

#### 3.4.2. Glycolic Acid as the Recycling C_2_

Glycolic acid is produced in measurable quantities in formose reactions [37], Miller spark-discharge experiments [38], and plays a potential role as a scaffold in oligopeptide-forming chemistry [39]. Could it play a role as the recycling C_2_ compound instead of ACE? In Figure 4, we plot the energy profile for glycolic acid (gold lines) relative to ACE (green lines) assuming the same reaction steps as the 3HP/4HB cycle. The net reaction is still similar to Equation (1) and the cycle overall exergonic at −45.03 kcal/mol.

The first CO_2_ addition forming tartronic acid is endergonic (6 kcal/mol), marginally less (by 1 kcal/mol) compared to the 3HP/4HB cycle. Three exergonic reactions follow: Two reductions lead to glyceric acid, which when dehydrated, converts into the enol of PYR. This provides a connection to the DC/4HB cycle (red line in Figure 2), providing an alternative starting point to having two early successive endergonic reactions. However, a switch to DC/4HB would mean glycolic acid is depleted rather than recycled; the net reaction as shown in Equation (4) would then be:

glycolic acid + 2 H_2_CO_3_ + 5 H_2_ → 2 ACE + 5 H_2_O (4)

with an overall free energy of −45.03 × 2 + 18.08 = −71.98 kcal/mol. (There is no cycle!) 

However, let us keep following the gold line. Reduction of PYR leads to lactic acid where it may potentially accumulate as a thermodynamic sink. The second addition of CO_2_ followed by a methyl shift leads to MAL. The analogous CO_2_ addition in the green line was only 3 kcal/mol endergonic (see Section 3.2.2), but for the gold line this is back to the higher 7–8 kcal/mol range. From MAL, reduction of a carboxylic acid to an aldehyde is marginally endergonic and an aldehyde-to-ketone isomerization (long gold line) analogous to SSA → AcACE avoids the over-reduction and formation of the 2-oxobutanoic acid enol. The final split produces ACE and regenerates glycolic acid.

However, if 2-oxobutanoic acid was produced, recall in the previous section that it is a less stable structural isomer of AcACE and part of the same “pool”. A keto-enol shift will convert it to AcACE which can split into two ACE. This would not recycle glycolic acid but provides a route to the 3HP/4HB cycle (green line in Figure 2), and illustrates one way such cycles crisscross and interconnect—a possible situation one might expect in a messier proto-metabolic milieu.

The kinetically challenging methyl-shift, however, remains problematic. Lactic acid could provide a way out. Unlike 3HP, ACR, or PRP, where the central carbon is the least positive and would be the site of CO_2_ addition (forming branched C_4_), in lactic acid the terminal methyl is the least positive carbon. If CO_2_ were to be added there, it could provide a route to unbranched MAL (*G_r_* = −48.14) and the DC/4HB cycle. This CO_2_ addition would only be endergonic by 4 kcal/mol, given that *G_r_*(lactic acid) is −52.03 kcal/mol, however it might still be kinetically challenging.

In fact, 3HP (*G_r_* = −51.71) could convert to lactic acid through a dehydration-rehydration via ACR—and this provides a tantalizing cycle which modifies the 3HP/4HB cycle by patching in a small part of the DC/4HB: ACE → MLN → MSA → 3HP → lactic acid → MAL → FUM → SUC → SSA → AcACE → split. Glycolic acid is not needed in this case.

#### 3.4.3. Ethanal or Glycolaldehyde (GA) as the Recycling C_2_

Having investigated glycolic acid as an oxidized cousin of ACE, we now examine the free energy profile starting of its reduced counterparts. The purple lines in Figure 4 are for ethanal as the recycling C_2_ species assuming the same reaction steps as the 3HP/4HB cycle; the net reaction is still Equation (1).

The first CO_2_ addition converts ethanal to MSA (a connection to the 3HP route!) and this oxidation is mildly endergonic (Δ*G* = +3 kcal/mol). Reduction of MSA to malonaldehyde is energy-neutral; reduction to 3-hydroxypropanal is exergonic as expected; dehydration to acrolein is energy-neutral; and then reduction to propanal is highly exergonic (analogous to ACR → PRP). The second CO_2_ addition to a branched C_4_ species, followed by the prebiotically questionable methyl shift, leads to SSA (in the 4HB route!). Isomerization to acetoaldehyde followed by splitting produces ACE and regenerates ethanal. The net reaction is Equation (1) and the net cycle is −45.03 kcal/mol. If instead, there is a switch to the 3HP/4HB cycle, ethanal is depleted rather than recycled in the net reaction as shown in Equation (5)

ethanal + 2 H_2_CO_3_ + 3 H_2_ → 2 ACE + 3 H_2_O (5)

with an overall free energy of −45.03 × 2 + 41.62 = −48.44 kcal/mol, but not a cycle.

One might also imagine (not shown), a rehydration of acrolein to form lactaldehyde, which perhaps might lead directly to SSA via CO_2_ addition to the terminal methyl. This might be possible if no reducing agent was present, but that is a challenge given the prior reduction steps.

**Figure 5:**
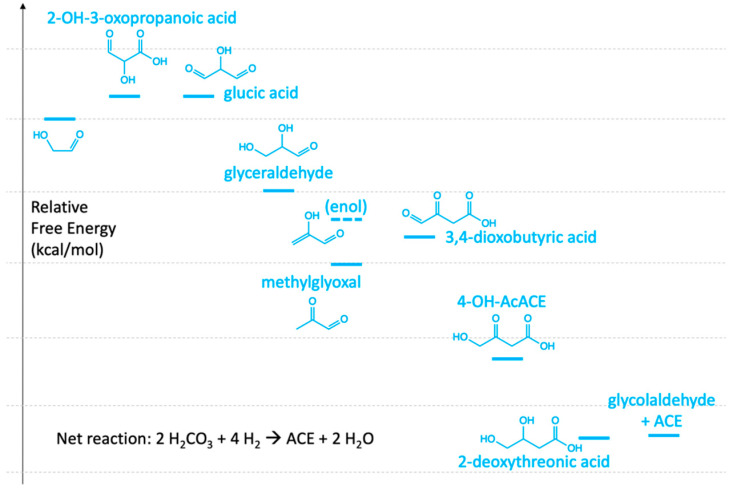
Potential cycle with glycolaldehyde as the C_2_ recycling species.

What if we start from GA? The first addition of CO_2_ leads to 2-OH-3-oxopropanoic acid (Δ*G* = +4 kcal/mol). Two reduction steps lead to glucic acid and glyceraldehyde (GLA), respectively. Dehydration leads to the enol form of methylglyoxal. If one were to add the second CO_2_ to methylglyoxal, kinetically it would be most feasible to form 3,4-dioxobutyric acid (Δ*G* = +4 kcal/mol), thereby avoiding branched C_4_ species (for the same reason as PYR → OXA). Reduction of the aldehyde and ketone to alcohols leads to 2-deoxythreonic acid, and then splitting produces ACE and regenerates GA. This last step is marginally endergonic (Δ*G* = +0.12 kcal/mol) but all other steps are exergonic except for the two CO_2_ additions. This free energy profile is shown in Figure 5.

This system has connections to CH_2_O oligomerization. GA is the recycling C_2_ species in the formose reaction, and GLA is the C_3_ species. One could imagine coupled cycles where GLA in the formose cycle can undergo dehydration to methylglyoxal, thereby accessing the cycle in Figure 5. We could also imagine including CH_2_O as an additional C_1_ food source, which would allow aldol addition to ACE to form 3HP, skipping MLN and MSA in the (green-line) 3HP/4HB cycle; or better yet transformations that utilize an aldol addition to a C_3_ to avoid forming branched C_4_ species. Changing the food source would also change the net cycle reaction and thermodynamics.

However, before we get too excited at the tantalizing possibilities, keep in mind that our speculations of potential cycles are based on just the thermodynamics; we have not quantitatively accounted for the kinetics, or reducing agents appearing at the appropriate time, or hydrating/dehydrating conditions, just to name a few issues.

#### 3.4.4. The Challenge of GLX

Earlier, we discussed the role of GLX as a high-energy intermediate, recycled in the 3HP bicycle pathway (Section 3.2.4). Could GLX play a role as the recycled C_2_ species in a simpler cycle such as the ones we have just examined? One problem: Adding the first equivalent of CO_2_ is significantly more endothermic, whether it produces oxomalonic acid (Δ*G* = +13 kcal/mol) with a +4 increase in oxidation state, or dioxopropanoic acid (Δ*G* = +14 kcal/mol) with concurrent addition of H_2_ and an overall +2 increase in oxidation state. If instead GLX is reduced in an earlier step before addition of CO_2_, this would be similar to the cases we have already examined.

Formation of GLX (*G_r_* = +0.22) is marginally endergonic from the reference molecules. Alternatives include the reaction of CO_2_ and H_2_CO, the two “food” molecules we have considered, which is +5 kcal/mol uphill; or HCO_2_H and CO which is +2 kcal/mol uphill (although CO would have to be generated prior). A prebiotic mixture might contain small amounts of GLX, but under reducing conditions, it would favorably convert into glycolic acid, ACE, or more reduced compounds.

However, as a high-energy reactant, GLX could drive thermodynamically favorable cycles. We previously saw (Section 3.2.4) that the downhill stretch of the 3HP bicycle has a free energy change of −37.77 kcal/mol. Stubbs et al. have experimentally designed a reverse TCA cycle analog that proceeds favorably in the absence of enzymes or metals as catalysts [40]. As shown in Table 3, our calculations show why this is thermodynamically favorable. The net chemical reaction as shown in Equation (6) is:

2 GLX → ACE + 2 H_2_CO_3_ + H_2_O (6)

The overall free energy change is −45.47 kcal/mol. Interestingly, the Jankowski group-additivity method [31] yields a similar overall value for this cycle, although there are differences in the individual steps. (eQulibrator does not have Δ_f_*G_i_’^0^* values for most of these “formates” so we are unable to make that comparison.) 

This cycle does not fix carbon overall nor is it meant to. Instead, it essentially reduces GLX to ACE while releasing units of CO_2_. GLX acts as an oxidizing agent, and this is its likely role in a proto-metabolic setting. With a *G_r_* similar to the reference compounds, it is the C_2_ equivalent of an “oxidizing” CO_2_ unit.

As a second example, we examine a non-enzymatic cycle designed experimentally by Muchowska et al. [41]. This cycle marries parts of the oxidative TCA and glyoxalate cycles, but by utilizing GLX as the oxidizing agent, the net cycle is now marginally exergonic (−0.22 kcal/mol), instead of being +45.03 kcal/mol endergonic as we saw in Section 3.1. The net reaction as shown in Equation (7) is:

GLX + 3 H_2_O → 2 H_2_CO_3_ + 2 H_2_ (7)

Individual steps in the cycle are shown in Table 4. The first half utilizes several compounds and reactions in common with *Stubbs* et al. [40], and we use the same names (from *Stubbs* et al.) in Table 3 rather than the different names used by Muchowska et al. (If comparing both papers, maloylformate = hydroxyketoglutarate; fumaroylformate = oxopentenedioate; isocitroylformate = oxalohydroxyglutarate.)

The cycle begins with PYR rather than OXA; Steps 2–4 are the same as Table 3; and Steps 7–9 are also found in the TCA cycle. While the main recycled compound is PYR, one equivalent of GLX is also recycled. (The two equivalents of GLX enter in Steps 1 and 4). From the quantum chemistry data, the very exergonic reductive Step 3 is balanced by the very endergonic oxidative Step 7. The Jankowski group additivity approach does not do as well here for the same reasons we saw in the TCA cycle. Where eQuilibrator values are available, our quantum calculations match up quite well. As in the previous example, GLX essentially acts a “high-energy” oxidizing agent allowing for exergonic steps.

## 4. Discussion

We are now ready to take a broader look at the overall thermodynamics of C_1_ to C_4_ species containing only carbon, hydrogen, and oxygen. We have arranged this map to emphasize redox states, inspired by Smith and Morowitz in their magisterial tome *The Origin and Nature of Life on Earth* [42]. We do not capture the intricacies they have investigated given the limited system size in our work, although our present analysis is influenced by Chapter 4 of their book.

**Figure 6:**
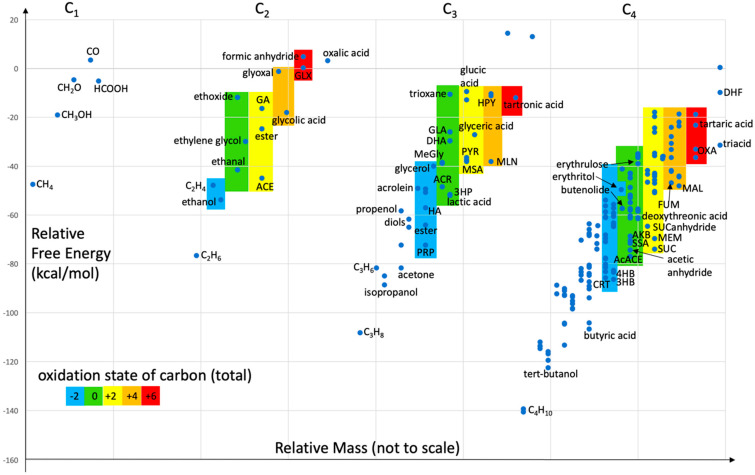
Overall thermodynamic redox map of C_1_ to C_4_ compounds (*G_r_* values in kcal/mol).

Our overall thermodynamic map is displayed in Figure 6. Recall that the reference molecules (with *G_r_* values of zero) are H_2_, H_2_O, H_2_CO_3_, and that our quantum chemical values are for neutral molecules in aqueous solution (zero ionic strength) under standard conditions at 298 K. The carbon “source” (CO_2_, or H_2_CO_3_ in aqueous solution) has +4 oxidation state; and our present map represents a reducing environment with H_2_ acting as the reductant. What features can we pick out under these conditions?

For C_1_ compounds, the most reduced compound CH_4_ (−4) is the most stable, followed by CH_3_OH (−2). Note that the aldehyde CH_2_O (0) and the acid HCOOH (+2) are of similar relative free energy. Carbon monoxide is the only compound higher in energy than the reference state. As size increases (towards the right in Figure 6), we see the same trends: alkanes, as the most reduced compounds, are the most stable; and relative free energies are less negative as oxidation state increases.

For C_2_ compounds, ethanol is more stable than ethene, ethanal is more stable than ethylene glycol, ACE is more stable than GA, and glycolic acid is more stable than glyoxal. The same trends hold for larger (C_3_ or C_4_) compounds of the same oxidation state: an OH group is more stable than having a C=C double bond; COOH is more stable than separate C=O and OH groups; C=O is more stable than a diol; having acid and alcohol groups is more stable than two C=O groups (recall the Cannizzaro products in Section 3.3). Additionally, the ester methylformate is less stable than its acid isomer ACE, and formic anhydride is less stable than its isomer GLX.

Looking at the most stable compounds across oxidation states for the C_2_ compounds, the series is ethane (−8), ethanol (−6), ethanal (−4), ACE (0), glycolic acid (+2), GLX (+4), oxalic acid (+6). Note that ACE is slightly more stable than ethanal, in the same way that HCO_2_H is slightly more stable than CH_2_O in the C_1_ series. For the C_3_ and C_4_ compounds, simple ketones are more stable than their corresponding aldehydes and acids; although the acid is still more stable than the aldehyde (PRP is slightly more stable than propanal and butyric acid is slightly more stable than butanal). This similarity in energy between the acid and aldehyde may be an important feature in proto-metabolism by allowing essentially energy-neutral and thus thermodynamically reversible redox transformations, possibly aided by primitive redox catalysts. We previously alluded to this feature: acids, aldehydes and ketones potentially form an equilibrating pool of molecules that may exert some control and regulation [36,37]. To this, we might add esters and anhydrides that might allow for the sequestering of acid metabolites.

In a column of molecules sharing the same oxidation state, carboxylic acids (if they can be formed) are always the most thermodynamically stable. In the C_3_ set for example, we have PRP (−2), lactic acid (0), MSA (+2), MLN (+4), and tartronic acid (+6). What are the most stable metabolites in the 3HP/4HB and the DC/4HB cycles? They are all acids and are among the most thermodynamically favored compounds in their respective columns. Once nature evolved specific enzymatic catalysts to reduce kinetic barriers, it seems to have maintained the primary use of thermodynamically stable compounds, the same ones you might expect to persist in a proto or pre-metabolic mixture.

An interesting metabolite not formally listed in the four core metabolic cycles is lactic acid, marginally more stable than its isomer 3HP (by 0.3 kcal/mol). We have speculated on its potential role in alternative (yet closely related) cycles in Section 3.4.4 but we do not yet know evolutionarily how it came to play its present role in extant metabolism. We should not discount it as an important player in a proto-metabolic system. Lactic acid’s close cousins, 3HP and ACR are part of the 3HB/4HB cycle.

In an organic compound with only a single functional group, a ketone is noticeably more stable than its aldehyde isomer. However, the free energy difference is smaller in more oxidized molecules and there are potential reversals: MSA is marginally more stable than PYR. MSA also has a stable enol thanks to its carbonyl group in the β-position with respect to the acid group. That is also why AcACE (in the 4HB route) is the most stable zero oxidation state C_4_ compound. Hence, if we imagined the simplest cycle utilizing the most stable acid molecules and CH_2_O as “food” to avoid oxidation state changes, the C_2_, C_3_, C_4_ candidates would be ACE, lactic acid (or 3HP), AcACE, respectively.

Could this be why ACE is the C_2_ species recycled in extant core metabolisms? It has the same zero oxidation state as GA (in the formose reaction), but is significantly more stable. Glycolic acid and GLX are favorably reduced to ACE thermodynamically, and ACE is also more stable than its reduced counterpart ethanal. Perhaps ACE combines the twin features of stability and simplicity. Furthermore, having an anionic form (in contrast to ethanal) might provide an anchor to a proto-metabolic mineral surface as suggested by Wachterhauser in his origin-of-life proposals [43], potentially presaging the ubiquitous role of phosphates (perhaps later) in the evolution of metabolism. AcACE also plays a key role as the splitting C_4_ species. Interestingly our calculations find that acetic anhydride is only marginally less stable than AcACE, and might play some role as a “pool” species if dehydrating conditions turn out to be important.

However, getting to AcACE is potentially tricky. We have proposed bypassing 4HB, CRT, 3HB, via a thermodynamically favorable aldehyde-to-ketone isomerization from SSA to avoid the late endergonic step. However, how do we get to SSA? At present, the most likely source is SUC, the only other molecule besides ACE to be present in all four core metabolisms shown in Figure 1. However, if SUC is being reduced to SSA, what stops reduction proceeding all the way to 4HB? It is unclear if there is a prebiotically plausible redox agent that will prevent reduction of the acid all the way to the alcohol, which may be why extant metabolism evolved this longer route. Additionally, how do you get to SUC? As previously discussed, routes involving MAL avoid the problem of converting a branched C_4_ into an unbranched species, but there are uphill steps to get to MAL. As alternatives, we speculated on routes involving lactic acid or methylglyoxal that get to MAL with minimal endergonicity. However, just because a reaction is thermodynamically favorable does not mean it is feasible in a prebiotic milieu.

We have discussed in Section 3.3 why an overall C_1_ + C_1_ → C_2_ reaction, embedded in a cycle is a more feasible way of incorporating food molecules into a growing metabolic system compared to the direct simple addition. With ACE as the C_2_ species, the net cycle is −45.03 kcal/mol. While two of its reduced cousins, ethanol and ethane, provide a more exergonic net reaction, both are less reactive and less likely to involve the rich carbonyl chemistry we have discussed. Ethanal could be a viable competitor (net cycle of −41.62 kcal/mol) but the lack of an anionic counterpart could be less robust for the anchoring advantages of selective surface chemistry. As to ACE’s oxidative cousins, glycolic acid might work (net cycle of −18.08 kcal/mol) although it favorably converts to ACE under reducing conditions (the carboxylate could be “protected” by surface attachment), and we have already discussed the challenge and potential role of GLX. These considerations may cement the key role played by ACE.

Extant metabolites participating in core cycles occupy a particular range of oxidation states depending on the size range of compounds involved. In the C_1_ to C_4_ group, these are mostly in the −2 to +4 oxidation states with OXA (+6) being just outside that group. The C_5_ and C_6_ compounds are mostly in the +2 to +6 range, with transient OXS at +8. Why these ranges? We can only speculate for now that there is a balance between adding oxidizing food equivalents (CO_2_ at +4) and reducing equivalents (H_2_) to form stable compounds in some optimal “Goldilocks” zone. Over-reduction to rock-bottom (overly stable) alkanes may limit chemistry. However, some reduction is needed to drive a thermodynamically favorable cycle.

Most of this section has focused on discussing variable oxidation states, but we would be remiss if we did not briefly discuss variable protonation states. Our calculated free energies use only neutral species (because of the poorer anion results). This necessarily means we lump together multiple species into a single entity even if it exists in more than one protonation state at significant concentration depending on the pH. Until we devise a protocol that provides better results compared to experiment for anionic species, these are an undifferentiated part of the aforementioned “pool” of equilibrating species.

## 5. Conclusions

We have explored the thermodynamics of uncatalyzed reductive TCA cycle and its chemical cousins by calculating the relative free energies of potential CHO metabolites in aqueous solution under standard conditions in the hope that it may shed light on how proto-metabolic systems are constructed and sustained. By focusing on simple cycles that incorporate C_1_ + C_1_ → C_2_ as the net reaction for building biomass, we considered why acetate is uniquely positioned as the key recycled compound, and tracked the energy changes of chemical transformations with an eye on oxidation state changes.

Could other food molecules change the free energy map? Yes. We would expect systematic shifts in our calculated thermodynamics. Would introducing co-factors (such as thioesters) change the energy profiles? Definitely. Such coupling reactions may provide ways around otherwise unfavorable endergonic reactions. Could other reference molecules be used, say if we employed a different redox couple? Certainly. Considering the extreme case of strongly oxidizing conditions with available O_2_ and no H_2_, our map in Figure 6 would flip. CH_4_ would be the least thermodynamically stable and CO_2_ would be rock-bottom on the scale. Would varying concentrations and dynamic flows of material and energy modify the situation? Most certainly. These questions and many more could be asked, but their answers are beyond the scope of the present work. Our group is actively exploring a number of these interesting rabbit-holes, several of which have been hinted at in this article, that build on the present baseline results.

This baseline provides a preliminary step to exploring the effect of temperature, pH, and different effective concentrations (activities) of the various solutes including the reference species. One could recalculate the relative free energy changes using Δ*G =* Δ*G_0_* + *RT* ln *Q*. Our baseline numbers provide the Δ*G_0_*. Solute activities (not equal to unity) and the effect of pH (via a [H^+^] term) in the reaction quotient *Q* would then result in different Δ*G* values. These only address equilibrium thermodynamics in a perturbative way. Temperature, pH, and solute activities are also expected to impact kinetics. Our group is working to calculate baseline activation barriers for reaction types in these cycles, although this requires the lengthy process of optimizing transition states.

Once baseline kinetics is established and we have made better estimates of the rate constants, we plan to incorporate both thermodynamic and kinetic parameters into a dynamic network model that tracks the fluxes of chemical species as they cycle through their myriad reactions. Our hope is that this will provide a preliminary model (with its attendant limitations) that represents the establishment of proto-metabolic cycles under non-equilibrium conditions, and we hope to provide a richer story in the future.

## Figures and Tables

**Table 1:** Δ*G* in kcal/mol for reactions in the oxidative TCA cycle with co-factors.

Step	Reaction	Textbook	Alberty	Quantum
1	acetyl-CoA + oxaloacetate + H_2_O → citrate + CoA	−7.5	−10.7	−4.5
2	citrate → cis-aconitate + H_2_O	+2.0	+2.0	+3.8
3	cis-aconitate + H_2_O → isocitrate	−0.5	−0.4	−3.0
4	isocitrate + NAD^+^ → α-ketoglutarate + CO_2_ + NADH	−2.0	−1.1	−0.3
5	α-ketoglutarate + NAD^+^ + H_2_O + P_i_ + ADP → succinate + CO_2_ + NADH + ATP	−8.0	−8.6	−10.5
6	succinate + FADenz_ox_ → fumarate + FADenz_red_	~0	0.0	N/A
7	fumarate → malate	−0.9	−0.9	−1.2
8	malate + NAD^+^ → oxaloacetate + NADH	+7.1	+6.9	+8.0
	***Net Cycle:***	***−9.8***	***−12.7***	***−7.7***

**Table 2:** Δ*G* in kcal/mol for reactions in the TCA cycle without co-factors.

Step	Reaction	Alberty	eQuil	Quantum (Neutral)	Quantum (Anions)	Jankowski
1	ACE + OXA → CIT	−0.81	−1.19	+2.49	+8.97	−0.71
2	CIT → CAC + H_2_O	+2.01	+2.80	+3.80	−3.34	+4.59
3	CAC + H_2_O → ISC	−0.43	+1.14	−3.02	+3.29	−3.17
4	ISC → OXS + H_2_	+19.04	+11.09	+17.22	+11.30	+8.02
5	OXS + H_2_O → AKG + H_2_CO_3_	−9.69	−2.31	−8.46	−7.51	−12.83
6	AKG + H_2_O → SUC + H_2_ + H_2_CO_3_	−6.75	−4.54	−7.98	−3.63	−4.16
7	SUC → FUM + H_2_	+25.38	+23.78	+27.12	+21.53	+19.12
8	FUM + H_2_O → MAL	−0.86	−0.74	−1.22	+4.74	−3.73
9	MAL → OXA + H_2_	+16.01	+14.60	+15.08	+14.26	+8.02
	***Net Cycle:***	***+43.90***	***+44.63***	***+45.03***	***+49.61***	***+15.15***

**Table 3:** Δ*G* in kcal/mol for the cycle designed by Stubbs et al.

Step	Reaction	Quantum	Jankowski
1	OXA + GLX + H_2_O → maloylformate + H_2_CO_3_	−7.12	−15.39
2	maloylformate → fumaroylformate + H_2_O	+0.78	+3.73
3	fumaroylformate + H_2_ → AKG	−26.88	−22.33
4	AKG + GLX → isocitroylformate	+0.30	−2.56
5	isocitroylformate → aconitoylformate + H_2_O	−0.30	+0.27
6	aconitoylformate + 2 H_2_O → CAC + H_2_CO_3_ + H_2_	−5.96	−4.46
7	CAC → CIT	−3.80	−5.49
8	CIT → OXA + ACE	−2.49	+0.71
	***Net Cycle:***	***−45.47***	***−45.12***

**Table 4:** Δ*G* in kcal/mol for the cycle designed by Muchowska et al.

Step	Reaction	eQuilibrator	Quantum	Jankowski
1	GLX + PYR → maloylformate	−3.09	−2.63	−0.75
2	maloylformate → fumaroylformate + H_2_O	not available	+0.78	+3.73
3	fumaroylformate + H_2_ → AKG	not available	−26.88	−22.33
4	AKG + GLX → isocitroylformate	not available	+0.30	−2.56
5	isocitroylformate + 2 H_2_O → ISC + H_2_CO_3_ + H_2_	not available	−9.28	−7.40
6	ISC → GLX + SUC	+1.39	+1.00	+5.08
7	SUC → FUM + H_2_	+23.78	+27.12	+19.12
8	FUM + H_2_O → MAL	−0.74	−1.22	−3.73
9	MAL → OXA + H_2_	+14.60	+15.08	+8.02
10	OXA + H_2_O → PYR + H_2_CO_3_	−5.16	−4.49	−14.64
	***Net Cycle:***	***N/A***	***−0.22***	***−15.46***

**Figure A1:**
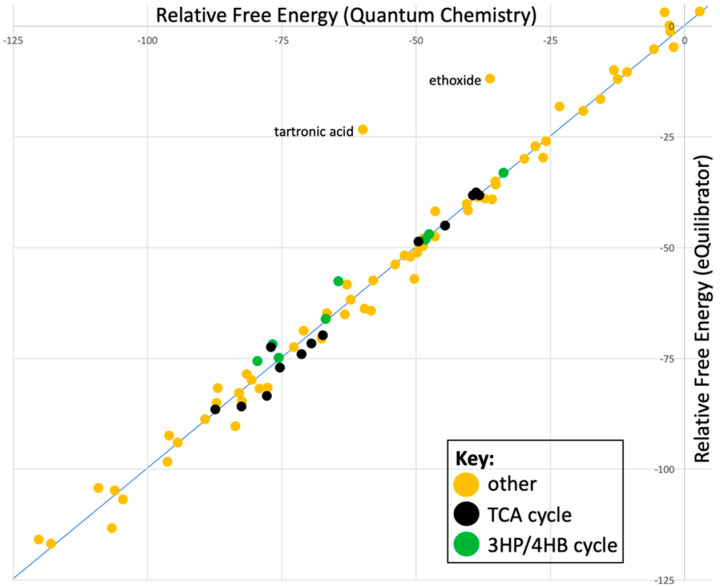
Comparison of quantum and available eQuilibrator *G_r_* values (kcal/mol).

## Data Availability

The data presented in this study are available in Supplementary Material.

## Supplementary Materials

The following are available online at https://www.mdpi.com/article/10.3390/life11101025/s1, Table S1: Comparison of Δ*G* (kcal/mol) for uncatalyzed oxidative TCA cycle reactions using M06-2X-D3 functional and the cc-pVDZ basis set; Table S2: Comparison of Δ*G* (kcal/mol) for uncatalyzed oxidative TCA cycle reactions using anions; Table S3: Comparison of Δ*G* (kcal/mol) for reductive 3HP/4HB Cycle; Table S4: Comparison of Δ*G* (kcal/mol) for additional reactions in DC/4HB cycle and 3HP bicycle; Table S5: Full set of compounds and Gr values (kcal/mol)

## Appendix A

Of the compounds in our full dataset of 223 compounds (see Table S5), 86 were found in the eQuilibrator database. Overall, from Figure A1, our quantum-calculated *G_r_* values closely match the experimentally-derived data. There are two outliers: ethoxide, a three-membered ring (likely due to different determination of ring strain); and tartronic acid, for no apparent reason we can surmise although we think the eQuilibrator value is wildly incorrect. Our quantum values for similar di-acids such as malic and tartaric acid match up well.

While a full list of all *G_r_* values, compound names, abbreviations, molecular formulae, and overall carbon oxidation states are found in Table S5, the truncated list in Table A1 below provides an easy reference to the compounds explicitly mentioned in the Results section. If “(hyd)” is included in the name, it means the hydrate is the most stable form. If “(enol)” is included in the name, the enol is the most stable form.

**Table A1:** *G_r_* values in kcal/mol for compounds in the Results section.

Name	Abbr.	Molecular Formula	Oxid. State	eQuilibrator *G_r_*	Quantum *G_r_*
formaldehyde (hyd)		CH2O	0	−2.08	−4.69
formicacid		CH4O2	2	−5.71	−5.23
ethanal		C2H4O	−2	−40.33	−41.62
acetic acid	ACE	C2H4O2	0	−44.63	−45.03
glylcolaldehyde	GA	C2H4O2	0	−15.68	−16.44
glycolic acid		C2H4O3	2	−23.33	−18.08
glyoxilic acid (hyd)	GLX	C2H4O4	4	−2.85	0.22
propanal		C3H6O	−4	−72.74	−72.43
acrolein		C3H4O	−2	−49.46	−49.18
propanoic acid	PRP	C3H6O2	−2	−77.04	−72.46
lactaldehyde (hyd)		C3H6O2	−2	−49.82	−51.06
3-OH-propanal		C3H6O2	−2	−48.77	−49.47
acrylic acid	ACR	C3H4O2	0	−49.53	−48.57
methylglyoxal (hyd)	MeGly	C3H4O2	0	−37.22	−38.93
malonaldehyde (enol)		C3H4O2	0	−38.63	−38.58
lactic acid		C3H6O3	0	−51.11	−52.03
3-OH-propanoic acid	3HP	C3H6O3	0	−52.18	−51.71
glyceraldehyde (hyd)	GLA	C3H6O3	0	−25.82	−25.99
di-OH-acetone	DHA	C3H6O3	0	−26.35	−29.71
pyruvic acid	PYR	C3H4O3	2	−38.87	−37.55
malonic semialdehyde (enol)	MSA	C3H4O3	2	−38.22	−38.20
glucic acid (enol)		C3H4O3	2		−12.87
glyceric acid		C3H6O4	2	−27.80	−27.12
malonic acid	MLN	C3H4O4	4	−39.42	−38.17
hydroxypyruvic acid	HPY	C3H4O4	4	−10.78	−10.33
2-OH-3-oxopropanoic acid (enol)		C3H4O4	4		−12.87
tartronic acid		C3H4O5	6	−12.53	−11.96
crotonic acid	CRT	C4H6O2	−2	−82.49	−85.80
3-butenoicacid		C4H6O2	−2		−80.19
succinaldehyde		C4H6O2	−2		−69.57
3-OH-butyric acid	3HB	C4H8O3	−2	−87.39	−86.46
4-OH-butyric acid	4HB	C4H8O3	−2	−77.78	−83.49
2-oxo-butanoic acid	AKB	C4H6O3	0	−70.92	−68.75
acetoacetic acid (enol)	AcACE	C4H6O3	0	−75.35	−77.03
succinic semialdehyde	SSA	C4H6O3	0	−69.51	−71.61
2-OH-but-3-enoic acid		C4H6O3	0		−54.93
erythrose		C4H8O4	0	−35.24	−35.00
erythrulose		C4H8O4	0	−35.84	−39.05
2-deoxythreonic acid		C4H8O4	0		−61.59
2-oxobutenoicacid		C4H4O3	2		−41.86
succinic acid	SUC	C4H6O4	2	−71.35	−74.04
methylmalonic acid	MEM	C4H6O4	2	−67.36	−69.75
2-OH-3-oxobutyric acid		C4H6O4	2		−50.97
4-OH-3-oxobutyric acid		C4H6O4	2		−50.38
fumaric acid	FUM	C4H4O4	4	−47.57	−46.92
3,4-dioxobutyric acid (enol)		C4H4O4	4		−33.28
malic acid	MAL	C4H6O5	4	−48.31	−48.14
oxaloacetic acid (enol)	OXA	C4H4O5	6	−33.71	−33.06
mesaconic acid	MSC	C5H6O4	2	−80.63	−79.87
citramalic acid	CTM	C5H8O5	2	−77.66	−81.55
methylmalic acid	MML	C5H8O5	2	−81.51	−78.51
alpha-ketoglutaric acid	AKG	C5H6O5	4	−66.81	−66.06
fumaroylformic acid		C5H4O5	6		−39.18
maloylformic acid		C5H6O6	6	−38.63	−39.96
cis-aconitic acid	CAC	C6H6O6	6	−76.73	−71.80
citric acid	CIT	C6H8O7	6	−79.53	−75.60
isocitric acid	ISC	C6H8O7	6	−75.59	−74.82
oxalosuccinic acid	OXS	C6H6O7	8	−64.50	−57.60
isocitroylformic acid		C7H8O8	8		−65.54
aconitoylformic acid		C7H8O8	8		−65.84
