# Supplementary material for: Thermodynamics of Potential CHO Metabolites in a Reducing Environment
Source: Life (Basel). 2021 Sep 29;11(10):1025. doi: 10.3390/life11101025 (PMC8537574; doi:10.3390/life11101025)
Supplement: Supplementary file 1 [file life-11-01025-s001.zip › life-1366544-supplementary.pdf]

life

MDPI

Supplementary Material

Thermodynamics of Potential CHO Metabolites in a Reducing Environment

Jeremy Kua *, Alexandra L. Hernandez and Danielle N. Velasquez

Department of Chemistry & Biochemistry, University of San Diego, San Diego, CA 92110, USA
* Correspondence: Correspondence: jkua@sandiego.edu

Part 1: Do newer "improved" DFT functionals give better results?

Previous work in our lab has compared our protocol (using the B3LYP functional) versus other functionals such as PBE or post–Hartree–Fock methods such as MP2. In these cases, B3LYP still gives better results when compared to available experimental results (which can be limited for the systems we have studied). One of the more recent robust functionals is M06–2X, but using this (along with the recommended D3 Grimme dispersion correction) in our protocol does not improve the B3LYP results as shown in Table S1. The recommended basis set to use with M06–2X at a similar level is cc–pVDZ but once again, the results are not an improvement over B3LYP//6–311G**. As stated in the Methods section of the main text, computational chemistry is about error–cancellation and our protocol at what seems an inferior older functional (B3LYP) works surprisingly well. One could attempt to dissect these results further to ascertain *why* the M06–2X do not perform as well, but that is beyond the scope of the present work.

Table S1. Comparison of ΔG (kcal/mol) for uncatalyzed oxidative TCA cycle reactions using M06–2X–D3 functional and the cc–pVDZ basis set.

Reaction	Alberty	B3LYP// 6–311G**	M06–2X–D3// 6–311G**	M06–2X–D3// cc–pVDZ
ACE + OXA → CIT	−0.81	+2.49	−10.13	−10.66
CIT → CAC + H_2O	+2.01	+3.80	+11.38	+15.80
CAC + H_2O → ISC	−0.43	−3.02	−11.41	−14.86
ISC → OXS + H_2	+19.04	+17.22	+27.63	+22.54
OXS + H_2O → AKG + H_2CO_3	−9.69	−8.46	−11.01	−4.74
AKG + H_2O → SUC + H_2 + H_2CO_3	−6.75	−7.98	−9.08	−10.97
SUC → FUM + H_2	+25.38	+27.12	+35.34	+31.66
FUM + H_2O → MAL	−0.86	−1.22	−8.84	−11.82
MAL → OXA + H_2	+16.01	+15.08	+25.58	+20.34
Net Cycle:	***+43.90***	***+45.03***	***+49.46***	***+37.29***

Part 2: Shouldn't all those carboxylic acids be carboxylates in solutions close to neutral pH?

Our present work is benchmarked against experimentally–derived (Alberty and eQuilibrator) at pH 7 where you would expect the majority of organic acids encountered to be predominantly in their anionic carboxylate form. As shown in Table 2 in the main text, the anions yield poorer results for the individual steps (although the net cycle is overall similar). In Table S2 below, we use the same abbreviations, but all acid groups are carboxylates. Here we also include M06–2X anion results. To repeat the adage, error–cancellation in our protocol works surprisingly well. One could attempt to dissect these results further to ascertain *why* the anions do not perform as well, but once again that's

beyond the scope of the present work. For B3LYP we found that using 6–311G**++ versus 6–311G**+ makes practically no difference. (For cc–pVDZ we only used ++)

Table S2. Comparison of Δ*G* (kcal/mol) for uncatalyzed oxidative TCA cycle reactions using anions.

Reaction	Alberty	B3LYP//<br>6–311G**+	M06–2X–D3//<br>cc–pVDZ++
ACE + OXA → CIT	−0.81	+8.97	−6.68
CIT → CAC + H_2O	+2.01	−3.34	+6.33
CAC + H_2O → ISC	−0.43	+3.29	−6.51
ISC → OXS + H_2	+19.04	+11.30	+19.78
OXS + H_2O → AKG + HCO_3(−)	−9.69	−7.51	−3.67
AKG + H_2O → SUC + H_2 + HCO_3(−)	−6.75	−3.63	+0.05
SUC → FUM + H_2	+25.38	+21.53	+26.78
FUM + H_2O → MAL	−0.86	−4.74	−3.51
MAL → OXA + H_2	+16.01	+14.26	+20.99
Net Cycle:	***+43.90***	***+49.60***	***+53.56***

Table S3 below compares the B3LYP//6–31G**+ anion calculations to the neutral molecule calculations alongside experimentally–derived eQuilibrator data and group–additivity Jankowski data. There is a good match between the neutral molecule quantum data and eQuilibrator, both for individual reactions and the net cycle. For the anion calculations, several steps show similar Δ*G* compared to the neutral case, but there are also notable differences (as large as 7 kcal/mol). The net cycle difference is ~5 kcal/mol. For the anions HCO_3(−) is used instead of H_2CO_3 in the two reactions requiring addition of a CO_2–equivalent.

Table S3. Comparison of Δ*G* (kcal/mol) for reductive 3HP/4HB Cycle.

Reaction	eQuilibrator	B3LYP (neutral)	B3LYP (anion)	Jankowski
ACE + H_2CO_3 → MLN + 2 H_2O	+5.21	+6.86	+4.02	+14.64
MLN + H_2 → MSA + H_2O	+1.20	−0.03	+4.06	−3.97
MSA + H_2 → 3HP	−13.96	−13.51	−13.63	−9.48
3HP → ACR + H_2O	+2.65	+3.14	−2.27	+12.99
ACR + H_2 → PRP	−27.51	−23.89	−24.59	−33.43
PRP + H_2CO_3 → MEM + H_2O	+9.68	+2.71	+4.55	+12.83
MEM → SUC	−3.99	−4.29	−2.58	+5.05
SUC + H_2 → SSA + H_2O	+1.84	+2.43	+4.39	−7.21
SSA +H_2 → 4HB	−8.27	−11.88	−11.04	−9.48
4HB → CRT + H_2O	−4.71	−2.31	−9.10	+1.92
CRT + H_2O → 3HB	−4.90	−0.66	+5.04	−3.73
3HB → AcACE + H_2	+12.04	+10.58	+8.70	+8.02
AcACE + H_2O → 2 ACE	−13.91	−14.18	−17.15	−3.30
Net Cycle:	***−44.63***	***−45.03***	***−49.60***	***−15.15***

The 3HP bicycle and the DC/4HB pathways overlap substantially with the TCA and 3HP/4HB pathways. Table S4 below includes unique reactions not found in Tables S2 and S3. For the anions HCO_3(−) is used instead of H_2CO_3 in the two reactions requiring addition of a CO_2–equivalent. For the ACE → PYR conversion, the Alberty Δ*G* is +8.06 kcal/mol. For the MAL → ACE + GLX conversion, Alberty Δ*G* is +1.46 kcal/mol. Alberty does not have values for MML, MSC and CTM. Overall, comparisons do not match up as well for reactions involving MML, MSC, CTM.

Table S4. Comparison of Δ*G* (kcal/mol) for additional reactions in DC/4HB cycle and 3HP bicycle.

Reaction	eQuilibrator	B3LYP (neutral)	B3LYP (anion)	Jankowski
ACE + H_2CO_3 + H_2 → PYR + 2 H_2O	+5.76	+7.48	+6.64	+7.40
PRP + GLX → MML	−1.62	−6.27	−0.54	+13.37
MML → MSC + H_2O	+0.81	−1.36	−5.01	+2.48
MSC + H_2O → CTM	−2.97	−1.68	−5.94	−11.89
CTM → PYR + ACE	−5.84	−1.03	−8.82	+0.71
MAL → ACE + GLX	+0.83	+3.33	+2.14	+0.75

Part 3: The Full Data Set

The full list of all G_r values, compound names, abbreviations, molecular formulae, and overall carbon oxidation states are found in Table S5 below. If "(hyd)" is included in the name, it means the hydrate is the most stable form. If "(enol)" is included in the name, the enol is the most stable form.

Table S5. Full set of compounds and G_r values (kcal/mol).

Name	Abbr.	Molecularformula	Oxid. State	eQuilibrator G_r	Quantum G_r
		C_1			
methane		CH4	−4	−46.49	−47.47
methanol		CH4O	−2	−18.88	−19.14
formaldehyde (hyd)		CH2O	0	−2.08	−4.69
carbon monoxide		CO	2	2.77	3.33
formicacid		CH4O2	2	−5.71	−5.23
		C_2			
ethane		C2H6	−6		−76.73
ethylene		C2H4	−4	−48.67	−47.91
ethanol		C2H6O	−4	−53.88	−53.78
ethanal		C2H4O	−2	−40.33	−41.62
ethoxide		C2H4O	−2	−36.24	−11.84
ethylene glycol	EG	C2H6O2	−2	−29.86	−29.99
methylformate		C2H4O2	0		−24.77
acetic acid	ACE	C2H4O2	0	−44.63	−45.03
glylcolaldehyde	GA	C2H4O2	0	−15.68	−16.44
glyoxal (hyd)		C2H6O4	2	−2.68	−1.23
glycolic acid		C2H4O3	2	−23.33	−18.08
formic anhydride		C2H2O3	4		4.74
glyoxilic acid (hyd)	GLX	C2H4O4	4	−2.85	0.22
oxalic acid		C2H2O4	6	−3.73	3.14
		C_3			
propane		C3H8	−8		−108.23
propene		C3H6	−6	−86.89	−81.69
propanol		C3H8O	−6	−87.18	−84.99
isopropanol		C3H8O	−6	−89.30	−88.72
propenol		C3H6O	−4	−62.92	−58.32
propanal		C3H6O	−4	−72.74	−72.43
acetone		C3H6O	−4	−79.19	−81.77
1,2−propanediol		C3H8O2	−4	−63.25	−65.05
1,3−propanediol		C3H8O2	−4	−62.20	−61.76

acrolein		C3H4O	−2	−49.46	−49.18
propanoic acid	PRP	C3H6O2	−2	−77.04	−72.46
lactaldehyde (hyd)		C3H6O2	−2	−49.82	−51.06
3−OH−propanal		C3H6O2	−2	−48.77	−49.47
OH−acetone	HA	C3H6O2	−2	−50.30	−57.06
methylacetate		C3H6O2	−2	−58.42	−64.22
glycerol		C3H8O3	−2	−40.59	−40.08
acrylic acid	ACR	C3H4O2	0	−49.53	−48.57
methylglyoxal (hyd)	MeGly	C3H4O2	0	−37.22	−38.93
malonaldehyde (enol)		C3H4O2	0	−38.63	−38.58
lactic acid		C3H6O3	0	−51.11	−52.03
3−OH−propanoic acid	3HP	C3H6O3	0	−52.18	−51.71
glyceraldehyde (hyd)	GLA	C3H6O3	0	−25.82	−25.99
di−OH−acetone	DHA	C3H6O3	0	−26.35	−29.71
trioxane		C3H6O3	0		−10.62
pyruvic acid	PYR	C3H4O3	2	−38.87	−37.55
malonic semialdehyde (enol)	MSA	C3H4O3	2	−38.22	−38.20
glucic acid (enol)		C3H4O3	2		−12.87
glycerosone		C3H4O3	2		−9.55
aceticformic anhydride		C3H4O3	2		−36.58
glyceric acid		C3H6O4	2	−27.80	−27.12
malonic acid	MLN	C3H4O4	4	−39.42	−38.17
hydroxypyruvic acid	HPY	C3H4O4	4	−10.78	−10.33
2−OH−3−oxopropanoic acid (enol)		C3H4O4	4		−12.87
dioxopropanoic acid		C3H2O4	6		14.35
tartronic acid		C3H4O5	6	−12.53	−11.96
oxomalonic acid		C3H2O5	8	0.45	12.93
		C_4			
butane		C4H10	−10		−139.36
isobutane		C4H10	−10		−140.56
but−1−ene		C4H8	−8		−112.02
butenecis		C4H8	−8		−114.69
butenetrans		C4H8	−8		−113.68
butenegem		C4H8	−8		−113.66
1−butanol		C4H10O	−8	−120.24	−115.92
1−isobutanol		C4H10O	−8	−117.99	−116.84
2−butanol		C4H10O	−8		−119.54
2−isobutanol		C4H10O	−8		−122.49
butadiene_trans		C4H6	−6	−95.98	−92.36
butadiene_cis		C4H6	−6		−88.88
butanal		C4H8O	−6	−106.09	−104.77
isobutanal		C4H8O	−6		−104.40
butanone		C4H8O	−6	−106.61	−113.27
but−1−en−3−ol		C4H8O	−6		−92.79
but−1−en−4−ol		C4H8O	−6		−90.16
but−2−enol_cis		C4H8O	−6		−91.06
but−2−enol_trans		C4H8O	−6		−92.93
but−2−enol_gem		C4H8O	−6		−93.04
isobutenol		C4H8O	−6		−93.03

tetrahydrofuran		C4H8O	−6		−93.04
1,2−butanediol		C4H10O2	−6		−95.32
1,3−butanediol		C4H10O2	−6		−96.34
1,4−butanediol		C4H10O2	−6		−93.09
2,3−butanediol		C4H10O2	−6	−96.26	−98.29
1,2−isobutanediol		C4H10O2	−6		−98.49
1,3−isobutanediol		C4H10O2	−6		−93.58
but−2−enal_trans		C4H6O	−4	−82.42	−84.61
but−2−enal_cis		C4H6O	−4		−82.00
but−2−enal_gem		C4H6O	−4		−83.45
methylvinylketone		C4H6O	−4		−86.80
dihydrofuran		C4H6O	−4		−70.38
butanoic acid		C4H8O2	−4	−109.14	−104.21
isobutanoic acid		C4H8O2	−4	−104.60	−106.81
2−OH−butanal		C4H8O2	−4		−81.49
3−OH−butanal		C4H8O2	−4		−84.06
4−OH−butanal		C4H8O2	−4		−80.99
2−OH−isobutanal		C4H8O2	−4		−84.79
3−OH−isobutanal		C4H8O2	−4		−80.63
1−OH−butanone		C4H8O2	−4		−87.46
3−OH−butanone		C4H8O2	−4	−83.69	−90.29
4−OH−butanone		C4H8O2	−4		−89.22
3,4−butenediol		C4H8O2	−4		−68.48
1,4butenediol_cis		C4H8O2	−4		−65.92
1,4−butenediol_trans		C4H8O2	−4		−68.01
isobutenediol		C4H8O2	−4		−67.62
ethylacetate		C4H8O2	−4	−94.42	−94.02
1,4−dioxane		C4H8O2	−4	−59.60	−63.73
1,2,3−butanetriol		C4H10O3	−4		−74.04
1,2,4−butanetriol		C4H10O3	−4		−71.73
1,2,3−isoproptriol		C4H10O3	−4		−74.04
1,3,3−isoproptriol		C4H10O3	−4		−68.62
furan		C4H4O	−2		−64.41
crotonic acid	CRT	C4H6O2	−2	−82.49	−85.80
isocrotonicacid		C4H6O2	−2		−83.26
3−butenoicacid		C4H6O2	−2		−80.19
methacrylicacid		C4H6O2	−2		−81.45
2−oxobutanal (enol)		C4H6O2	−2		−68.18
3−oxobutanal (enol)		C4H6O2	−2		−78.02
succinaldehyde		C4H6O2	−2		−69.57
3−oxobutanone		C4H6O2	−2		−77.74
methylmalonaldehyde (enol)		C4H6O2	−2		−71.07
OH−methylacrolein_gem		C4H6O2	−2		−58.73
OH−methylacrolein_cis		C4H6O2	−2		−56.39
OH−methylacrolein_trans		C4H6O2	−2		−60.74
C4ald_2OH_alkene		C4H6O2	−2		−53.95
C4ket_OH_alkene		C4H6O2	−2		−61.21
gammabutyrolactone		C4H6O2	−2		−83.21
2−OH−butyric acid		C4H8O3	−2	−82.99	−82.79

3−OH−butyric acid	3HB	C4H8O3	−2	−87.39	−86.46
4−OH−butyric acid	4HB	C4H8O3	−2	−77.78	−83.49
2,3−diOH−butanal		C4H8O3	−2		−60.42
2,4−diOH−butanal		C4H8O3	−2		−57.18
3,4−diOH−butanal		C4H8O3	−2		−59.22
1,3−diOH−butanone		C4H8O3	−2		−64.26
1,4−diOH−butanone		C4H8O3	−2		−63.28
dihydrofuran−3−one		C4H8O3	−2		−64.30
3,4−diOH−butanone		C4H8O3	−2		−64.83
2,3−diOH−isobutanal		C4H8O3	−2		−60.62
3,3−diOH−isobutanal		C4H8O3	−2		−55.68
erythritol		C4H10O4	−2	−48.72	−49.61
vinylglyoxal		C4H4O2	0		−41.21
butenolide		C4H4O2	0	−58.02	−57.35
2−oxo−butanoic acid	AKB	C4H6O3	0	−70.92	−68.75
acetoacetic acid (enol)	AcACE	C4H6O3	0	−75.35	−77.03
succinic semialdehyde	SSA	C4H6O3	0	−69.51	−71.61
2−methyl−3−oxopropanoic acid (enol)		C4H6O3	0	−67.60	−70.49
OH−methacrylicacid		C4H6O3	0		−57.61
2−OH−but−3−enoic acid		C4H6O3	0		−54.93
gamma−OH−crotonic acid		C4H6O3	0		−60.36
gamma−OH−isocrotonic acid		C4H6O3	0		−57.50
3−OH−2−oxobutanal		C4H6O3	0		−42.93
4−OH−2−oxobutanal		C4H6O3	0		−43.23
2−OH−3−oxobutanal (enol)		C4H6O3	0		−51.88
4−OH−3−oxobutanal		C4H6O3	0		−48.80
2−OH−4−oxobutanal		C4H6O3	0		−44.86
1−OH−3−oxobutanone (enol)		C4H6O3	0		−52.89
2−OH−butyrolactone		C4H6O3	0		−58.10
3−OH−butyrolactone		C4H6O3	0		−61.23
acetic anhydride		C4H6O3	0		−74.44
erythrose		C4H8O4	0	−35.24	−35.00
erythrofuranose		C4H8O4	0		−36.27
threose		C4H8O4	0	−35.19	−35.81
threofuranose		C4H8O4	0		−36.57
erythrulose		C4H8O4	0	−35.84	−39.05
2−CHO−glycerol		C4H8O4	0		−34.98
4−deoxythreonic acid		C4H8O4	0		−61.25
3−deoxythreonic acid		C4H8O4	0		−58.48
3,4−diOH−butyricacid		C4H8O4	0		−61.59
2,3−diOH−isobutyricacid		C4H8O4	0		−61.19
3,3−diOH−isobutyricacid		C4H8O4	0		−57.65
2−oxobutenoicacid		C4H4O3	2		−41.86
4−oxobutenoic_trans acid		C4H4O3	2		−46.57
4−oxobutenoic_cis acid		C4H4O3	2		−43.34
succinic anhydride		C4H4O3	2	−66.60	−64.75
succinic acid	SUC	C4H6O4	2	−71.35	−74.04
methylmalonic acid	MEM	C4H6O4	2	−67.36	−69.75
3−OH−2−oxobutyric acid		C4H6O4	2		−43.51

4−OH−2−oxobutyric acid		C4H6O4	2		−43.76
2−OH−3−oxobutyric acid		C4H6O4	2		−50.97
4−OH−3−oxobutyric acid		C4H6O4	2		−50.38
2−OH−4−oxobutyric acid		C4H6O4	2		−45.75
3−OH−4−oxobutyric acid		C4H6O4	2		−47.01
2−OH−3−oxoisopropanoic acid		C4H6O4	2		−44.25
3−OH−3−oxoisopropanoic acid (enol)		C4H6O4	2		−45.60
3,4−diOH−2−oxobutanal		C4H6O4	2		−18.01
2,4−diOH−3−oxobutanal		C4H6O4	2		−24.38
2,3−diOH−4−oxobutanal		C4H6O4	2		−19.74
1,4−diOH−3−oxobutanone (enol)		C4H6O4	2		−26.19
erythronolactone		C4H6O4	2		−35.82
threolactone		C4H6O4	2		−34.75
erythronic acid		C4H8O5	2		−36.10
threonic acid		C4H8O5	2		−37.03
maleic anhydride		C4H2O3	4		−36.03
fumaric acid	FUM	C4H4O4	4	−47.57	−46.92
maleicacid		C4H4O4	4	−46.45	−41.79
methylenemalonic acid		C4H4O4	4		−42.15
2,3−dioxobutyric acid		C4H4O4	4		−27.98
2,4−dioxobutyric acid (enol)		C4H4O4	4		−30.69
3,4−dioxobutyric acid (enol)		C4H4O4	4		−33.28
glycolide		C4H4O4	4		−24.19
malic anhydride		C4H4O4	4		−36.52
malic acid	MAL	C4H6O5	4	−48.31	−48.14
methyl−OH−malonic acid		C4H6O5	4		−44.13
methyltartronic acid		C4H6O5	4		−44.49
3,4−diOH−2−oxobutanoic acid		C4H6O5	4		−18.66
2,4−diOH−3−oxobutanoic acid		C4H6O5	4		−23.70
2,3−diOH−4−oxobutanoic acid		C4H6O5	4		−22.02
oxaloacetic acid (enol)	OXA	C4H4O5	6	−33.71	−33.06
aldehydemalonic acid (enol)		C4H4O5	6		−36.52
tartaric acid		C4H6O5	6	−59.95	−23.29
isotartaric acid		C4H6O5	6		−18.87
diOH−fumaraldehyde (enol)		C4H6O5	6		−8.53
methaneTCacid		C4H4O6	8		−31.51
diOH−fumaric acid (enol)		C4H4O6	8	−13.20	−9.89
OH−aldehydemalonic acid		C4H4O6	8		0.33
dioxosuccinic acid		C4H2O6	10		22.07
		C_5			
mesaconic acid	MSC	C5H6O4	2	−80.63	−79.87
citramalic acid	CTM	C5H8O5	2	−77.66	−81.55
methylmalic acid	MML	C5H8O5	2	−81.51	−78.51
alpha−ketoglutaric acid	AKG	C5H6O5	4	−66.81	−66.06
fumaroylformic acid		C5H4O5	6		−39.18
maloylformic acid		C5H6O6	6	−38.63	−39.96
		C_6			
cis−aconitic acid	CAC	C6H6O6	6	−76.73	−71.80
citric acid	CIT	C6H8O7	6	−79.53	−75.60

isocitric acid	ISC	$C6H8O7$	6	−75.59	−74.82
oxalosuccinic acid	OXS	$C6H6O7$	8	−64.50	−57.60
		C_7			
isocitroylformic acid		$C7H8O8$	8		−65.54
aconitoylformic acid		$C7H8O8$	8		−65.84
